# Lecithin exerts direct lipid-lowering and antioxidant cytoprotective effects *in vitro*: implications for laying hen fatty liver hemorrhagic syndrome

**DOI:** 10.3389/fphys.2026.1848092

**Published:** 2026-07-20

**Authors:** Juan Xiong, Jinchao Zhang, Huiwen Mo, Bingjie Wang, Li Zhang, Shouneng Shi, Rui Fang, Junlong Zhao

**Affiliations:** 1 National Key Laboratory of Agricultural Microbiology, College of Veterinary Medicine, Huazhong Agricultural University, Wuhan, Hubei, China; 2 Centree Bio-Tech (Wuhan) Co., Ltd., Wuhan, Hubei, China; 3 Veterinary Research Institute, Xinjiang Academy of Animal Sciences (Animal Clinical Medical Research Center, Xinjiang Academy of Animal Sciences), Urumqi, China; 4 The Twins (group) Co., Ltd., Nanchang, Jiangxi, China

**Keywords:** cytoprotective mechanism, fatty liver hemorrhagic syndrome, laying hen, lecithin, steatosis

## Abstract

**Background:**

Fatty liver hemorrhagic syndrome (FLHS) is a prevalent metabolic disorder in laying hens that severely compromises physiological health and egg production. Lecithin, a vital phospholipid complex, is known for its lipotropic and cytoprotective properties; however, whether its preventive effects against FLHS act directly on the liver or rely on systemic shifts remains elusive. This study aimed to investigate the direct cytoprotective role and molecular mechanisms of lecithin using an isolated cell-based model of FLHS.

**Methods:**

An *in vitro* steatosis model was established by treating Leghorn male hepatoma (LMH) cells with exogenous free fatty acids (FFAs). Lipid accumulation and redox status were assessed by quantifying intracellular triglycerides (TG), superoxide dismutase (SOD) activity, and reduced glutathione (GSH) content. The expression of key lipogenic genes, including acetyl-CoA carboxylase (*ACC*) and stearoyl-CoA desaturase 1 (*SCD1*), was validated via qPCR. Furthermore, transcriptomic sequencing (RNA-seq) was integrated with bioinformatic analysis to identify differentially expressed genes (DEGs) and functional signaling pathways.

**Results:**

Lecithin supplementation significantly attenuated FFAs-induced TG accumulation (p < 0.05) and restored cellular redox homeostasis by increasing SOD activity and GSH levels (p < 0.05). In normal hepatocytes, lecithin downregulated the expression of *ACC* and *SCD1* (p < 0.01). Under steatotic conditions, lecithin specifically suppressed *SCD1* mRNA levels (p < 0.05). Transcriptomic profiling identified 397 DEGs between the FFA and FFA + lecithin groups. Functional enrichment analysis revealed that these DEGs were primarily involved in oxidoreductase activity and the arachidonic acid metabolism pathway, particularly focusing on cyclooxygenase and prostaglandin-endoperoxide synthase activities.

**Conclusion:**

This study demonstrates that lecithin possesses the capacity to directly protect hepatocytes by attenuating lipid accumulation and enhancing antioxidant defenses, suggesting that its preventive role against FLHS is driven, at least in part, by direct hepatic action rather than relying solely on systemic metabolism. Furthermore, we hypothesize that lecithin may potentially offer additional protection through indirect anti-inflammatory actions. These findings provide a robust theoretical foundation for elucidating the direct hepatoprotective mechanisms of lecithin, contributing to a clearer understanding of its role in FLHS prevention and supporting its scientific dietary supplementation in laying hens, particularly during the peak and late laying periods.

## 1 Introduction

Eggs serve as high-quality protein sources for human nutrition (Réhault Godbert et al., 2019). The substantial demand for eggs necessitates large-scale layer farming (Chen et al., 2025). However, the intensive cage housing systems employed in such large-scale production are associated with a significantly higher incidence of nutritional and metabolic diseases, such as fatty liver hemorrhagic syndrome (FLHS), in laying hens (Lay et al., 2011). Statistically, FLHS—a nutrition-related metabolic disorder caused by dysregulated lipid metabolism—is responsible for approximately 40% of deaths in laying hens, a proportion that increases to 74% in caged systems (Shini et al., 2018). Clinically, affected laying hens often display increased body weight, and an enlarged, pale, and friable liver frequently presents hemorrhagic spots (Hansen and Walzem, 1993). Therefore, preventing and controlling FLHS has become a critical and urgent issue in the layer industry (San et al., 2023).

Lecithin typically refers to a class of lipid mixtures composed primarily of phospholipids (Küllenberg et al., 2012). Plant-derived phospholipids are amphipathic molecules characterized by a hydrophilic head group consisting of a phosphate moiety attached to the sn-3 position of glycerol, and two hydrophobic fatty acid tails esterified at the sn-1 and sn-2 positions (Li-Beisson et al., 2013). By virtue of this structural feature, phospholipids not only serve as essential components of the lipid droplet and lipoprotein coat, playing a critical role in lipid transport and metabolism, but also act as precursors of lipid mediators involved in cell signaling, functioning as second messengers (Kakisaka et al., 2012). Additionally, phospholipids exhibit antioxidant capacity by chelating pro-oxidant metal ions. Their unsaturated fatty acids also enhance membrane fluidity, which critically influences the function of cells, lipid droplets, and lipoproteins (Klingler et al., 2016). Consequently, lecithin effectively regulates various membrane-dependent cellular functions. By integrating these anti-inflammatory, antioxidant, and lipid-modulating properties, lecithin ultimately improves overall lipid and lipoprotein profiles (Robert et al., 2020).

Dietary lecithin supplementation can effectively improve the production performance of laying hens, regulate lipid metabolism, and prevent FLHS. For example, one study revealed that soybean lecithin alleviated hepatic pathological changes and the aberrant expression of the apolipoproteins apoA-I and apoB-100 induced by FLHS (Song et al., 2017). The efficacy of lecithin varies with its source, which is attributable to differences in phospholipid and fatty acid compositions (Blesso, 2015). A study on the effects of dietary safflower phospholipids demonstrated their capacity to reduce hepatic triglycerides and serum cholesterol in laying hens without adverse effects (An et al., 1997). Furthermore, several studies have confirmed that longterm lecithin supplementation improves egg production efficiency, ameliorates blood and hepatic lipid profiles, enhances hepatic antioxidant capacity, and optimizes liver and blood parameters associated with FLHS, thereby supporting liver health (Hu et al., 2022; Yang et al., 2017). Despite these prior findings, it remains elusive whether the preventive efficacy of lecithin against FLHS is mediated by a direct protective action on the liver or indirectly driven by systemic secondary metabolic shifts. To address this knowledge gap, the present study established an *in vitro* high-fat model to explicitly exclude systemic confounding factors. By isolating the cellular environment, we aimed to ascertain whether lecithin exerts a direct cytoprotective effect on hepatocytes and to preliminarily explore its underlying molecular mechanisms. Ultimately, these insights are expected to provide a robust theoretical foundation for the scientific dietary supplementation of lecithin to prevent FLHS in layer production.

## 2 Materials and methods

### 2.1 Cell culture

Leghorn male hepatoma (LMH) cells were used in this study. LMH cells were cultured in DMEM/F-12 basal medium (SH30023.01, Cytiva, Hangzhou, China) supplemented with 10% fetal bovine serum (FBS) (B265994, Aladdin, Shanghai, China). The cells were maintained as adherent monolayers in T25 culture flasks (707001, NEST Biotechnology Co. LTD, Shanghai, China) at 37 °C in a humidified atmosphere containing 5% CO_2_.

### 2.2 Cell viability detection

LMH cells were passaged at approximately 80% confluence (usually within 2 days) with EDTA-trypsin (T1304, Solarbio, Beijing, China) for 1 minute. The cells were subsequently seeded into 96-well plates (701012, NEST Biotechnology Co. LTD, Shanghai, China) at a density of 1×10^5^ cells/mL. After 24 hours, the complete medium (DMEM/F-12 with 10% FBS) was replaced with fresh medium containing various concentrations of phosphatidylcholine (0, 20, 30, 40, or 50 µM) (L426059, Aladdin, Shanghai, China), with six replicate wells for each treatment. After 48 hours of incubation, 10 µL of CCK-8 reagent (A311-01/02, Vazyme, Nanjing, China) was added to each well. Blank control wells were also included. The plates were then incubated for 2 hours at 37 °C in the dark. Finally, the absorbance at 450 nm was measured via a microplate reader (Spark^®^, Tecan, Switzerland).

### 2.3 Liver oil red O staining

LMH cells were seeded into 6-well plates (703012, NEST Biotechnology Co., Ltd., Shanghai, China) at a density of 3×10^5^ cells/mL and incubated for 24 hours. The control group and FFA group were treated with DMEM/F-12 medium and a PO mixture (palmitic acid: oleic acid = 75 µM:150 µM) (P0500, Sigma Aldrich, Shanghai, China; O431503, Aladdin, Shanghai, China), respectively. Each treatment was performed in triplicate, with 2 mL of medium per well. After 48 hours of incubation, lipid accumulation was assessed via Oil Red O staining (C0157M, Beyotime, Shanghai, China) following the manufacturer’s instructions.

### 2.4 Biochemical measurements

Twenty-four hours after seeding in 6-well plates, LMH cells were assigned to four treatment groups (in triplicate): the control group (treated with the DMEM/F-12 medium), FFA group (treated with the DMEM/F-12 medium and PO mixture), FFA + lecithin group (treated with the PO mixture and 30 µM phosphatidylcholine), and lecithin group (treated with 30 µM phosphatidylcholine alone). After 48 hours of treatment, the cells from each well were collected using cell scrapers (710011, NEST Biotechnology Co., Ltd., Shanghai, China) in 50 µL of isopropanol (I119459; Aladdin, Shanghai, China). After ultrasonic lysis, the intracellular levels of triglyceride (TG) (S0219S, Beyotime, Shanghai, China), total cholesterol (TC) (S0211S, Beyotime, Shanghai, China), and free fatty acids (FFA) (S0215S, Beyotime, Shanghai, China), as well as the activities of related enzymes, were measured following the manufacturer’s instructions. For the determination of total protein (TP) content (A045-4-2, Nanjing Jiancheng Biotechnology Research Institute Co., Ltd., Nanjing, China), superoxide dismutase (SOD) activity (A001-3-2, Nanjing Jiancheng Biotechnology Research Institute Co., Ltd., Nanjing, China), reduced glutathione (GSH) content (A006-2-1, Nanjing Jiancheng Biotechnology Research Institute Co., Ltd., Nanjing, China), malondialdehyde (MDA) content (A003-1-2, Nanjing Jiancheng Biotechnology Research Institute Co., Ltd., Nanjing, China), and total antioxidant capacity (TAOC) (A015-2-1, Nanjing Jiancheng Bio-technology Research Institute Co., Ltd., Nanjing, China), cells were collected in 250 µL of normal saline (IN9000, Solarbio, Beijing, China) as described above (with isopropanol replaced by saline) and analyzed according to the kit protocols. All measurements were performed in triplicate to ensure reliability.

### 2.5 RT–PCR

All primers used were obtained from the literature and are listed in **Table 1**. Following the same treatment as described for biochemical measurements, LMH cells from all four groups were individually lysed in 200 µL of ultrapure water for nucleic acid extraction. Using an automatic nucleic acid extraction system (VNP-32P, Vazyme, China), 200 µL of sample per well was processed following the kit protocol (RM201-02, Vazyme, Nanjing, China), and the extracted nucleic acids were temporarily stored at 4 °C. Reverse transcription was conducted via EasyScript^®^ One-Step gDNA Removal and cDNA Synthesis SuperMix (AE311-02, TransGen Biotech, Beijing, China), and the reaction mixture was prepared according to the manufacturer’s instructions. The reaction procedure was set as follows: incubation at 25 °C for 10 min, followed by 42 °C for 30 min, and finally 85 °C for 5 min.

**Table 1:** The primers used for quantitative RT–PCR ^1^.

Name	Forward prime sequence (5′-3′)	Reverse prime sequence (5′-3′)
FAS	CCTGGCATCCTATTATATTGACT	TATGCTGCCACAAAGGAATGAGA
ACC	TGGACTGGAAAACGTCTCGG	CACAGGTACGCCTTTACCGT
SCD1	AGCTACACTGCCCCTGCGGA	AGCCTTTAAATCACTCAGGTCCA
CPT1	GGAGAACCCAAGTGAAAGTAATGAA	TGGAAACGACATAAAGGCAGAA
β-actin	TGCGTGACATCAAGGAGAAG	ATACCCAAGAAAGATGGCTGGAA

^1^ *FAS*, fatty acid synthase; ACC, acetyl-CoA carboxylase; SCD1, stearoyl-CoA desaturase 1; *CPT1*, carnitine palmitoyl transferase 1.

### 2.6 Quantitative real-time PCR

Fluorescence detection was performed with 2× Taq Pro Universal SYBR qPCR Master Mix (Q712-02, Vazyme, Nanjing, China). The quantitative real-time PCR (qPCR) reaction was performed in a total volume of 20 µL, containing 10 µL of 2× Taq Pro Uni-versal SYBR qPCR Master Mix, 0.8 µL each of forward and reverse primers, 2 µL of template cDNA, and 6.4 µL of ddH2O. The qPCR cycling conditions were as follows: initial denaturation at 95 °C for 30 s, followed by 40 cycles of denaturation at 95 °C for 10 s and annealing/extension at 60 °C for 30 s. The melt curve stage was automatically generated by the instrument (FMR3, Vazyme, China). The relative expression of target genes was calculated via the 2^–ΔΔCt^ method (Livak and Schmittgen, 2001), with β-actin used as the endogenous reference for normalization. All the experiments were conducted with three in-dependent replicates.

### 2.7 Transcriptome analysis

The cells from the FFA and FFA+lecithin groups were collected with 1 mL of RNAiso Plus (9108, Takara Bio, Beijing, China) as described above. We sent the resulting RNA samples which directly dissolved in RNAiso Plus reagent to Personal Bio-technology Co., Ltd. (Shanghai, China) for transcriptome analysis. Subsequently, the returned data were analyzed using the Personal Gene Cloud online analysis platform (https://www.genescloud.cn/). The mRNA expression levels were normalized to fragments per kilobase of transcript per million mapped reads (FPKM). Differentially expressed genes (DEGs) were identified using a threshold of p < 0.05 and |log2fold-change|> 1.5. In addition, the sign of the log2fold-change indicates whether a gene is up regulated or down regulated, whereas the p value is used to determine whether the difference is statistically significant. Significantly enriched GO terms and KEGG pathways were identified using a threshold of corrected p value < 0.05.

### 2.8 Statistical analysis

The data are presented as the means ± standard deviations of three independent biological replicates. Homogeneity of variances was confirmed by Levene’s test (p>0.05) before ANOVA. All the statistical analyses were performed via GraphPad Prism software. Differences between groups were assessed by one-way analysis of variance (ANOVA), and a p value of less than 0.05 was considered statistically significant.

## 3 Results

### 3.1 Effects of lecithin and PO mixtures

**Figure 1:**
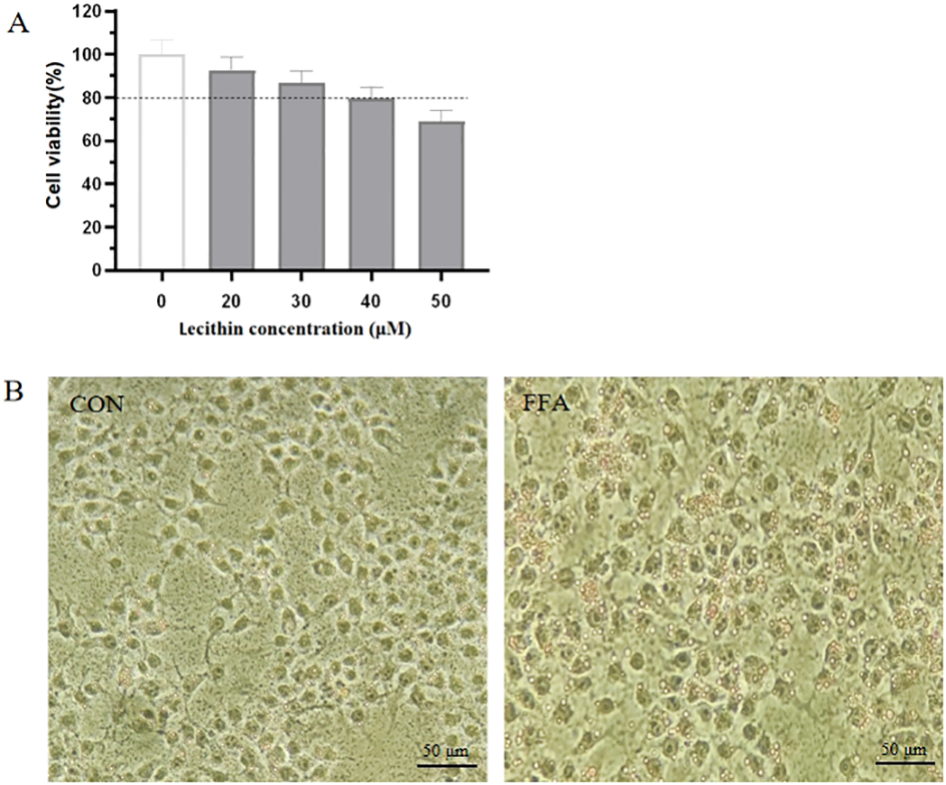
Effects of the lecithin and PO mixture. **(A)** Effects of lecithin on LMH cell viability. **(B)** LMH Oil Red O Staining. The data represent mean ± SEM (n = 6 per group). CON, control group; FFA, FFA group.

On the basis of the results of the CCK-8 assay, 30 µM was identified as a well-tolerated concentration of lecithin in LMH cells, with over 80% cell viability maintained after 48 hours of treatment (**Figure 1A**). Given its low cytotoxicity, 30 µM was selected for subsequent experiments. As shown in **Figure 1B**, compared with the control group, the FFAs-treated group presented a significant increase in the number of intracellular red-stained lipid droplets. This observation indicates that the FFAs induced significant lipid accumulation in LMH cells, indicating the successful establishment of a suitable *in vitro* steatosis model for subsequent experiments.

### 3.2 Changes in lipid metabolism-related indicators

**Figure 2:**
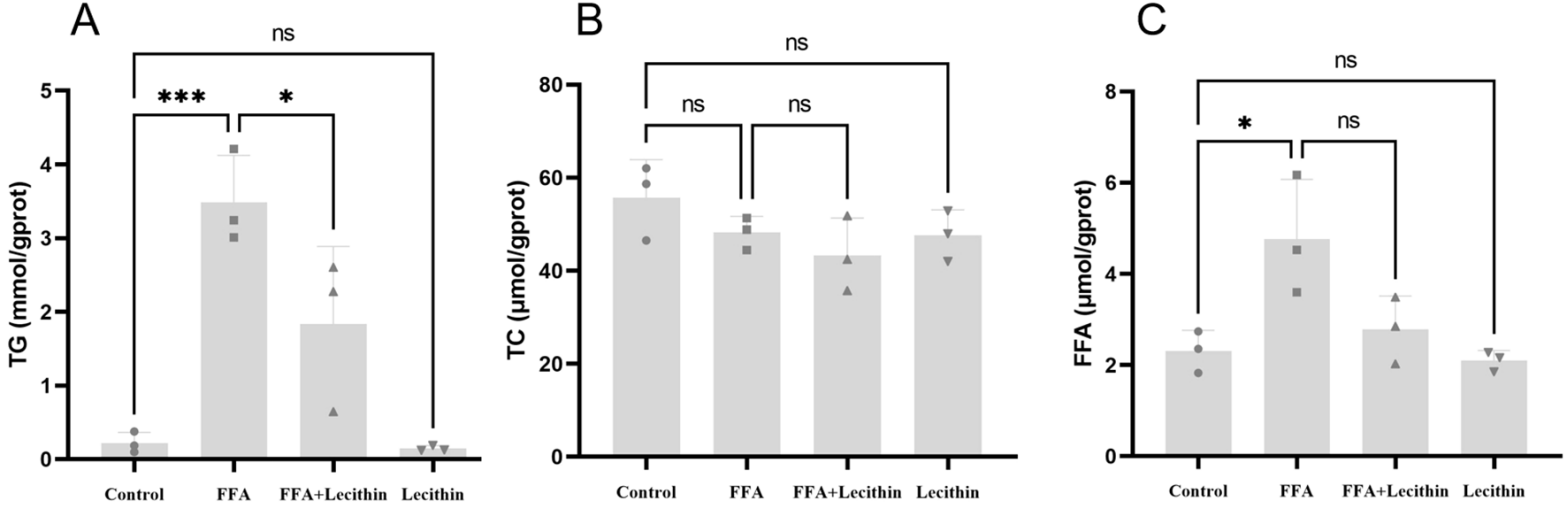
Changes in lipid metabolism-related indicators. **(A)** The changes of triglyceride (TG) concentration. **(B)** The changes of total cholesterol (TC) concentration. **(C)** The changes of free fatty acid (FFA) concentration. The data represent mean ± SEM (n = 3 per group). Differences were determined by 1-way ANOVA. *indicates P < 0.05; **indicates P < 0.01; ***indicates P < 0.001.

Intracellular levels of triglyceride (TG), total cholesterol (TC), and free fatty acid (FFA) were measured using commercial kits. Compared with the control group, the FFA group presented a significant increase in TG (p < 0.001; **Figure 2A**), confirming the validity of the model. Compared with the FFA group, the FFA + Lecithin group showed a significant decrease in TG content (p < 0.05; **Figure 2A**). In contrast, TC levels remained unchanged across all groups (**Figure 2B**). Although FFA induction increased the cellular FFA level (p < 0.05; **Figure 2C**), lecithin supplementation did not significantly change this parameter.

### 3.3 Changes in antioxidant parameters

**Figure 3:**
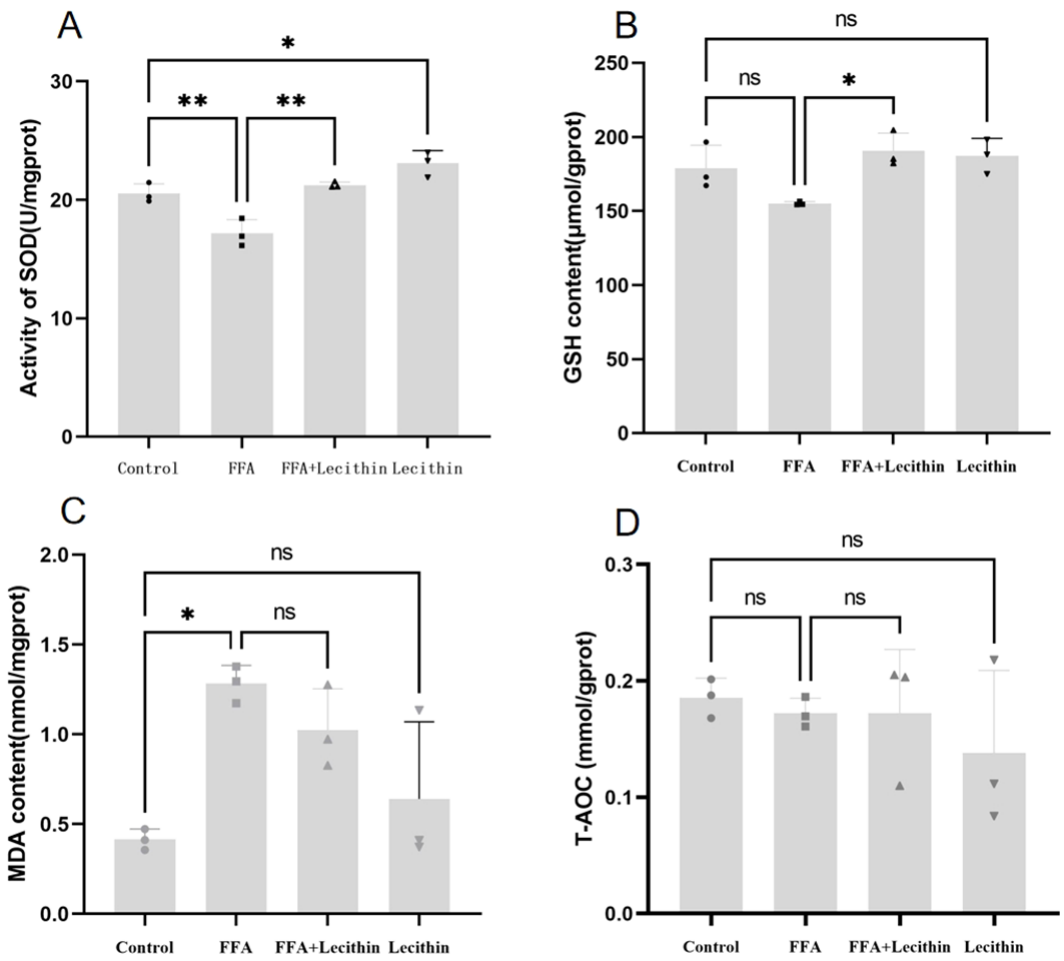
Changes in antioxidant parameters. **(A)** Changes in superoxide dismutase (SOD) activity. **(B)** Changes in reduced glutathione (GSH) content. **(C)** Changes in malondialdehyde (MDA) con-tent. **(D)** Changes in total antioxidant capacity (TAOC). The data represent mean ± SEM (n = 3 per group). Differences were determined by 1-way ANOVA. * indicates P < 0.05; ** indicates P < 0.01.

The activities of superoxide dismutase (SOD), reduced glutathione (GSH) content, malondialdehyde (MDA) content, and total antioxidant capacity (TAOC) level were measured using corresponding commercial kits, respectively. As shown in **Figure 3A**, SOD activity was significantly lower in the FFA group than in the control group (p < 0.01). Supplementation with lecithin significantly increased SOD activity in the FFA + lecithin group relative to that in the FFA group (p < 0.01). Lecithin alone also significantly increased SOD activity compared with the control (p < 0.05), indicating that lecithin can increase total SOD activity in LMH cells. Compared with the FFA group, the FFA + lecithin group presented significant increases in GSH levels (p < 0.05; **Figure 3B**). **Figure 3C** showed that the MDA content was significantly greater in the FFA group than in the control group (p < 0.05). However, lecithin treatment did not significantly affect the MDA levels in any group (p > 0.05). Finally, TAOC was not significantly changed by lecithin in any treatment group (p > 0.05; **Figure 3D**).

### 3.4 Changes in LMH lipid metabolism-related genes

**Figure 4:**
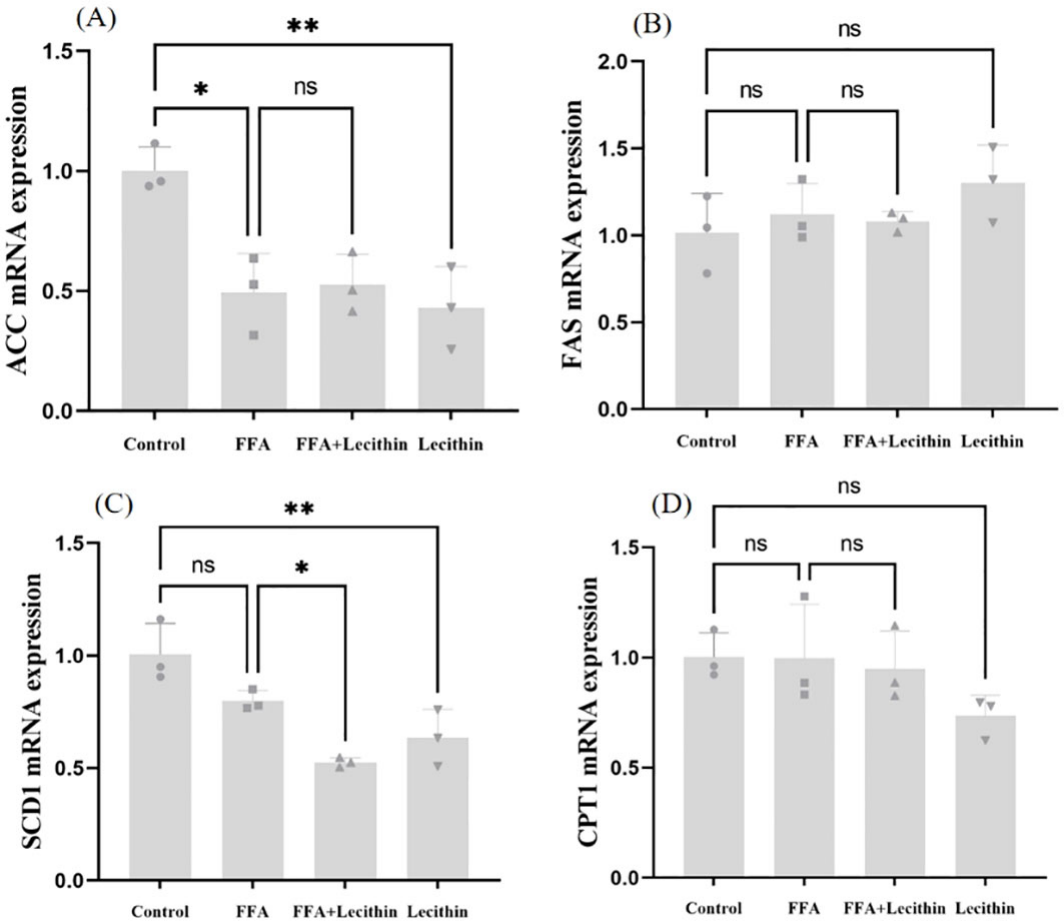
Changes in LMH lipid metabolism-related genes. **(A)** The changes of *ACC* mRNA ex-pressions. **(B)** The changes of *FAS* mRNA expressions. **(C)** The changes of *SCD1* mRNA expressions. **(D)** The changes of *CPT1* mRNA expressions. The data represent mean ± SEM (n = 3 per group). Differences were determined by 1-way ANOVA. *ACC*, acetyl-CoA carboxylase; *FAS*, fatty acid synthase; *SCD1*, stearoyl-CoA desaturase 1; *CPT1*, carnitine palmitoyl transferase 1. *indicates P < 0.05; **indicates P < 0.01.

Lecithin significantly downregulated the transcriptional level of acetyl-CoA carboxylase (*ACC*) in LMH (**Figure 4A**). Compared with the control group, the lecithin group presented highly significant reductions in *ACC* expression (p < 0.01). Notably, the FFA group exhibited a significant decrease in *ACC* expression relative to the control group (p < 0.05) and no statistically significant difference was observed between the FFA + Lecithin group and the FFA group (p > 0.05). Lecithin had no significant effect on the transcriptional level of fatty acid synthase (*FAS*) (p > 0.05; **Figure 4B**). As shown in **Figures 4C, stearoyl**-CoA desaturase 1 (*SCD1*) expression was significantly lower in the FFA + lecithin group than in the FFA group (p < 0.05). Moreover, the lecithin group alone demonstrated a highly significant reduction in *SCD1* transcription relative to that of the control group (p < 0.01). Like its lack of effect on *FAS*, lecithin did not significantly alter the transcriptional level of carnitine palmitoyl transferase 1 (*CPT1*) (p > 0.05; **Figure 4D**).

### 3.5 Transcriptome sequencing data analysis

**Figure 5:**
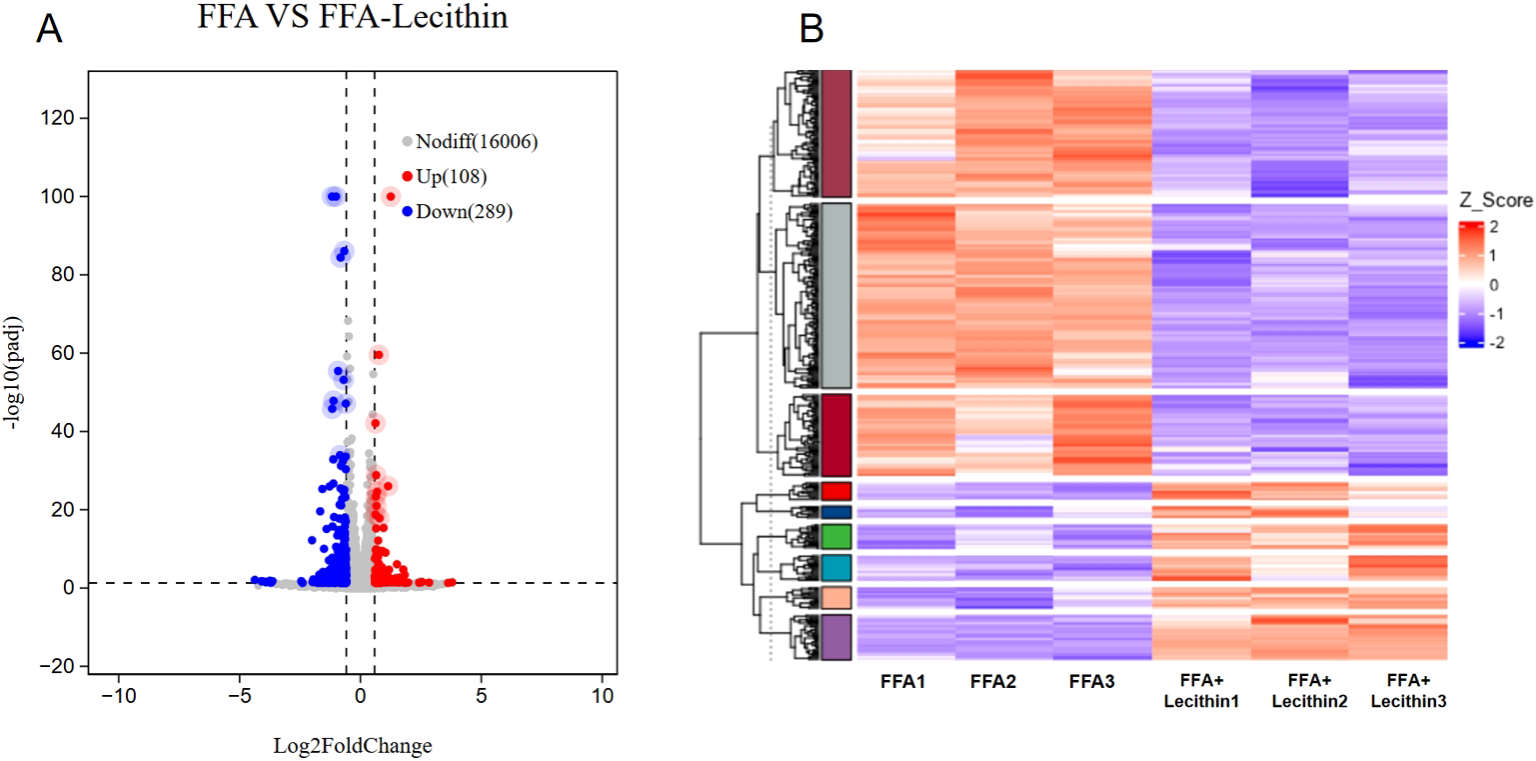
Transcriptome sequencing data analysis. **(A)** Volcano plot of differentially expressed genes. Red and blue represent upregulated and downregulated genes, respectively. **(B)** Hierarchical cluster analysis of differentially expressed genes. Blue represents downregulated genes, and red represents upregulated genes.

**Figure 6:**
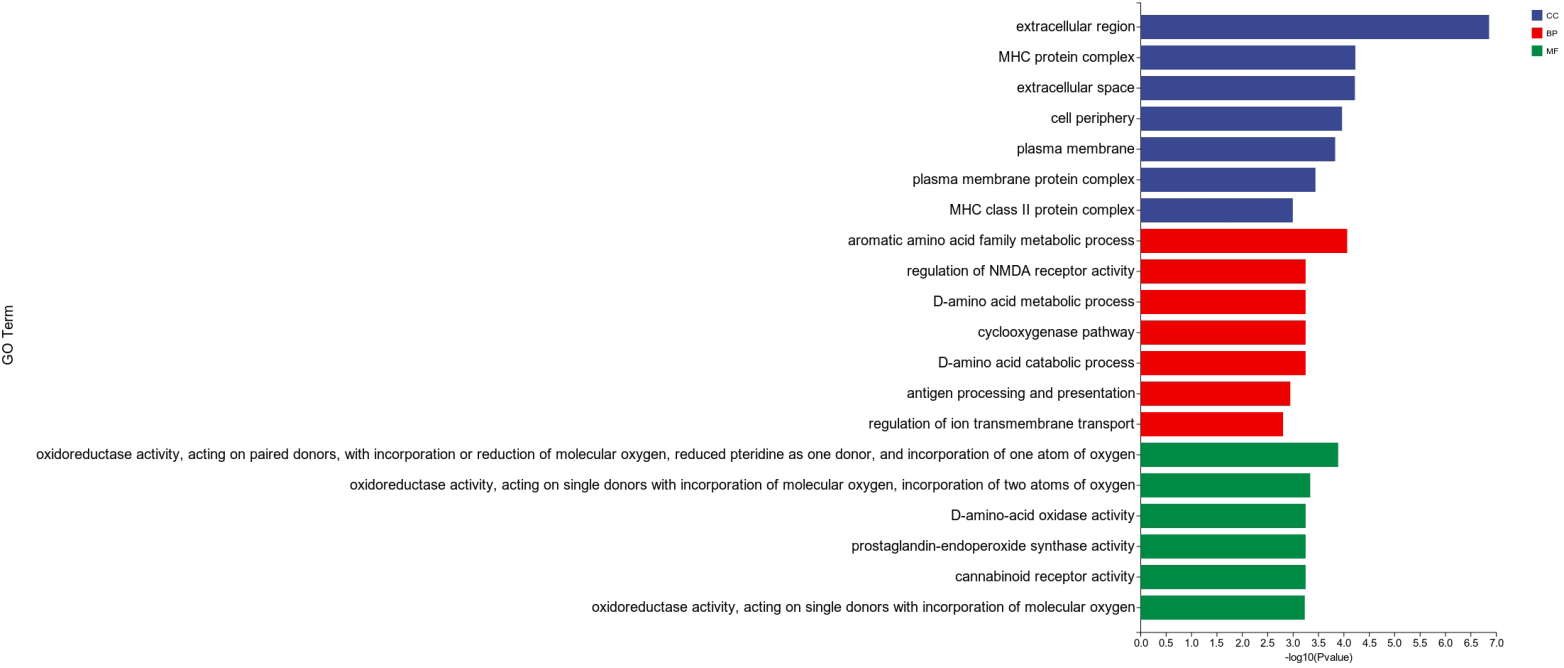
GO enrichment analysis of the differentially expressed genes. A threshold of p < 0.05 and |log2fold-change|> 1.5 were used to select significant GO categories. The blue bars indicate the number of DEGs enriched in cellular component, and the red bars indicate the number of DEGs in the biological process. the green bars indicate the number of differentially expressed genes (DEGs) enriched in molecular function.

**Figure 7:**
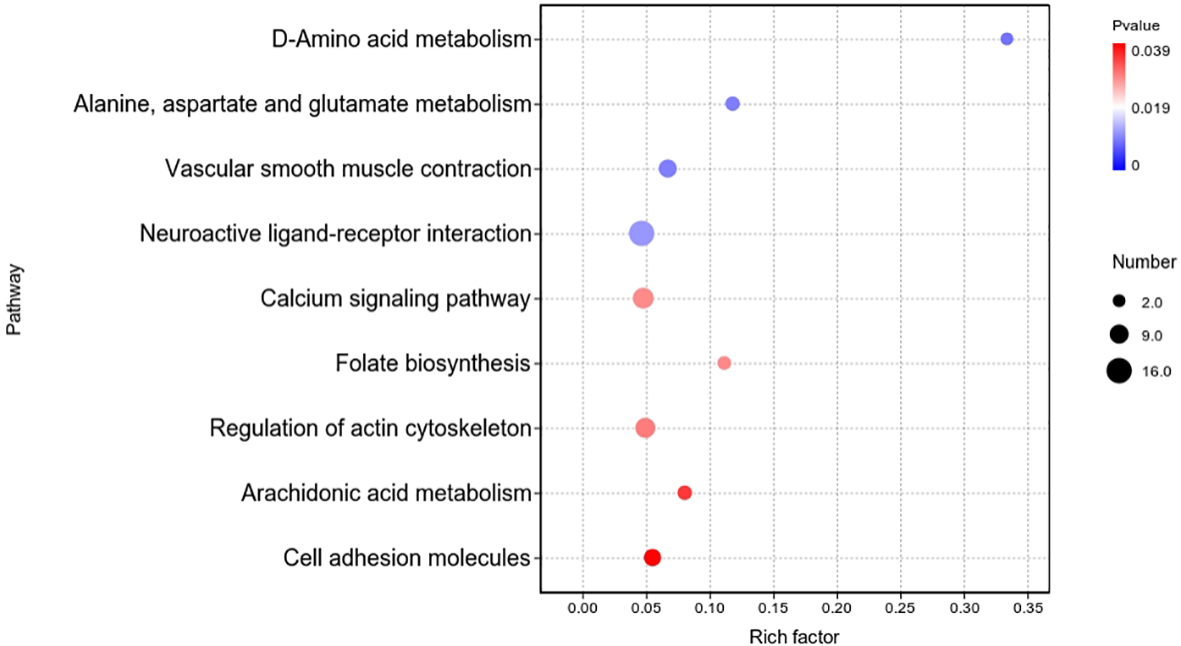
KEGG pathway functional enrichment analysis of the DEGs. The top 9 significantly enriched pathways were list. The dot size is proportional to the number of enriched DEGs per pathway; color intensity indicates the significance level.

A total of 397 differentially expressed genes (DEGs) were identified, comprising 108 upregulated and 289 downregulated genes (**Figure 5A**). As illustrated in **Figure 5B**, hierarchical clustering analysis revealed the distinct expression patterns of these DEGs between the two treatment groups while also reflecting a high degree of expression similarity among samples within the same group. Further functional annotation via GO analysis revealed the top 20 significantly enriched terms in the categories of biological process (BP), molecular function (MF), and cellular component (CC) (**Figure 6**). These enriched terms encompassed various biological aspects, including the extracellular region, plasma membrane, cyclooxygenase pathway, prostaglandin-endoperoxide synthase activity, oxidoreductase activity, and cannabinoid receptor activity. KEGG pathway enrichment analysis indicated that DEGs were moderately enriched in the arachidonic acid metabolism pathway (**Figure 7**). With respect to the lipid metabolism-related genes identified from the transcriptomic analysis, the expression of *SCD1* was significantly lower in the FFA + lecithin group than in the FFA group (log2-fold change < 0, P < 0.05). Conversely, the transcript levels of *FAS*, *CPT1*, and *ACC* were not significantly affected (P > 0.05) (**Table 2**).

**Table 2:** Transcriptomic results of lipid metabolism-related genes^1^.

Gene	Log2Foldchange	Pval	Regulation
SCD1	-0.1371	0.0334	Nodiff
FAS	-0.06593	0.6778	Nodiff
CPT1	-0.009193	0.7997	Nodiff
ACC	-0.004429	0.9905	Nodiff

^1^ *SCD1*, stearoyl-CoA desaturase 1; *FAS*, fatty acid synthase; *CPT1*, carnitine palmitoyl transferase 1; *ACC*, acetyl-CoA carboxylase. Log_2_ fold change: Positive values indicate increased expression, and negative values indicate decreased expression. p−value: Represents the statistical significance of the expression difference. Regulation: Indicates the type of regulation (up−regulated or down−regulated). Nodiff: Denotes no significant differential expression.

## 4 Discussion

Inducing cellular steatosis via the use of exogenous free fatty acids, typically at an oleic acid to palmitic acid ratio of 2:1, is an established and effective method for promoting intracellular lipid accumulation (Cheng et al., 2024; Luan et al., 2026). The steatosis model was validated by Oil Red O staining and elevated TG content. Although TC and FFA levels remained unchanged—a discrepancy from some prior studies (Ding et al., 2024; Yao et al., 2022)—this outcome falls within the expected spectrum of metabolic responses to lipid overload. As suggested by lipidomic analyses, initial metabolic stress primarily drives the synthesis and accumulation of TG, whereas cholesterol fluctuations may require longer induction periods or different stressors (Liu et al., 2025; You et al., 2023). Against this backdrop of TG-dominant accumulation, lecithin supplementation significantly reduced the intracellular TG content in FFAs-induced cells (p < 0.05), despite considerable variability within the group. This lipid-lowering efficacy aligns robustly with extensive *in vivo* evidence demonstrating that dietary lecithin significantly decreases hepatic TG in laying hens (Hu et al., 2022; Song et al., 2017) and mammalian models (Lu et al., 2022). Reflecting the clinical depletion of hepatic phosphatidylcholine in hens with FLHS (You et al., 2023), our *in vitro* results provide direct evidence that lecithin acts autonomously on hepatocytes to exert a lipid-lowering cytoprotective effect, independent of systemic metabolic modulations.

Excessive lipid accumulation induces oxidative stress, a key driver of hepatic injury and FLHS in laying hens (Miao et al., 2025; Sun et al., 2024). Our findings demonstrate that lecithin co-treatment effectively bolsters the hepatocyte antioxidant defense system by elevating SOD activity and GSH levels. This intrinsic antioxidant property is further supported by *in vitro* studies in other cell types; for instance, phosphatidylcholine treatment in HepG2 cells significantly suppressed Reactive Oxygen Species (ROS) generation and oxidative damage (Choi et al., 2022). This reinforces our observation that lecithin or its components can directly stimulate enzymatic defenses (Beheshti Moghadam et al., 2021; Hu et al., 2022). While *in vivo* studies consistently report reduced MDA and increased TAOC with lecithin (Wu et al., 2023), our *in vitro* model showed no significant changes in these markers. This discrepancy is mechanistically rational: lecithin primarily replenishes damaged membranes and alleviates organelle stress as a structural phospholipid, rather than acting as a direct chemical scavenger (Mak and Shekhar, 2024). Furthermore, the reliance on a single 48-hour time point represents a static snapshot of a dynamic process. The lack of consecutive monitoring may have failed to capture the specific metabolic window during which MDA clearance and macroscopic TAOC recovery become statistically detectable. In summary, we infer that lecithin exerts a direct protective effect on hepatocytes by enhancing their intrinsic antioxidant capacity.

To further explore whether lecithin influences lipid metabolism at the transcriptional level, we evaluated the expression of key lipid metabolism genes (*ACC*, *FAS*, *CPT1*, and *SCD1*). In several previous *in vitro* studies, steatotic hepatocytes typically exhibited a significant upregulation of *ACC*, *FAS*, and *SCD1*, accompanied by a downregulated or unchanged *CPT1* profile (Ding et al., 2024; Luan et al., 2026; Miao et al., 2025). Conversely, our model presented a divergent expression pattern, notably characterized by a significant downregulation of *ACC* in the FFAs-induced group. This discrepancy may be attributable to time-course limitations and dosage differences (Muta et al., 2022; You et al., 2023). For instance, the single 48-hour detection snapshot likely captured a late negative feedback phase rather than the initial compensatory upregulation. Nevertheless, despite the temporal complexities in the steatotic model, our study still observed that lecithin effectively downregulated the expression of *ACC* and *SCD1* in normal hepatocytes, which strongly aligns with previous findings (Mak and Shekhar, 2024; Osipova et al., 2022; Yang et al., 2025). This indicates that lecithin can influence lipid metabolism-related genes in hepatocytes, thereby affirming its lipid-lowering capacity.

The transcriptomic analysis corroborated the expression trends of the evaluated lipid metabolism genes, thereby validating the reliability of the sequencing data. Notably, the transcriptomic results revealed a significant enrichment in “oxidoreductase activity,” further reinforcing the inference that lecithin can enhance the antioxidant capacity of hepatocytes. Crucially, the transcriptomic profile highlighted the “cyclooxygenase (COX) pathway” and “arachidonic acid (ARA) metabolism.” Dysregulation of ARA metabolism has been identified as a key lipidomic signature in avian FLHS (You et al., 2023), a metabolic axis known to drive the synthesis of pro-inflammatory mediators via COX activation under stress conditions in poultry (Guo et al., 2025). The anti-inflammatory potential of lecithin, as indicated by these transcriptomic findings, aligns with observations in other hepatocyte models where phosphatidylcholine significantly suppresses pro-inflammatory cytokines (e.g., TNF-α, IL-6) and inhibits the activation of NF-κB signaling (Choi et al., 2022; Sanchez et al., 2024). Furthermore, exogenous phosphatidylcholine has been shown to stabilize cellular membranes and effectively restrict the activity of phospholipase A2 (PLA2), the primary enzyme responsible for releasing free ARA from the lipid bilayer (Gundermann et al., 2011, 2016). Therefore, we boldly speculate that lecithin may also exert direct hepatoprotective effects through anti-inflammatory actions. Specifically, by replenishing the membrane phospholipid pool, exogenous phosphatidylcholine could inhibit the abnormal hyperactivation of PLA2, thereby restricting the release of free ARA and dampening COX-mediated inflammation.

In summary, the present *in vitro* study demonstrates that lecithin can act directly on hepatocytes to exert targeted lipid-lowering and antioxidant cytoprotective effects. Furthermore, we propose a transcriptomic-derived hypothesis that lecithin may provide additional hepatoprotection through potential anti-inflammatory mechanisms. However, several limitations of this study must be acknowledged. First, the singular FFAs-induced steatosis model cannot fully recapitulate the complete, multi-stage pathogenesis of clinical fatty liver disease. Second, the reliance on a single lecithin concentration and a single intervention time point restricts the comprehensive understanding of dose-response and temporal dynamics. Future research should employ multi-stage lipid overload models and utilize primary avian hepatocytes to more accurately dissect these cellular protective mechanisms. Ultimately, comprehensive *in vivo* experiments are warranted to validate these hypotheses and evaluate the true contribution of lecithin’s direct hepatoprotective effects to the prevention of FLHS in laying hens.

## 5 Conclusions

In conclusion, using an *in vitro* FFAs-induced LMH cell model, we demonstrated that lecithin effectively attenuates intracellular triglyceride accumulation and inferred its capacity to enhance the intrinsic antioxidant defense system of hepatocytes. Furthermore, based on transcriptomic profiling, we hypothesize that lecithin may secondarily modulate arachidonic acid metabolism and the cyclooxygenase pathway, thereby offering potential indirect regulation against lipid-induced inflammatory responses. We cautiously conclude that the *in vivo* preventive efficacy of lecithin against FLHS is driven, at least in part, by these direct cytoprotective actions—namely, targeted lipid reduction and antioxidant enhancement—directly at the hepatic level. These insights provide a robust theoretical foundation for elucidating the direct hepatoprotective mechanisms of lecithin, ultimately offering solid *in vitro* evidence to support its scientific and quantitative supplementation in the diets of laying hens, particularly during the peak and late laying periods.

## Data Availability

The data presented in the study are deposited in the Zenodo repository, accession number 10.5281/zenodo.21130715.
