# Supplementary material for: Lecithin exerts direct lipid-lowering and antioxidant cytoprotective effects in vitro: implications for laying hen fatty liver hemorrhagic syndrome
Source: Front Physiol. 2026 Jul 20;17:1848092. doi: 10.3389/fphys.2026.1848092 (PMC13429444; doi:10.3389/fphys.2026.1848092)

Each experiment was performed independently in triplicate, and the results of the three replicates for key experiments are shown below.

1. Triglyceride (TG) measurement


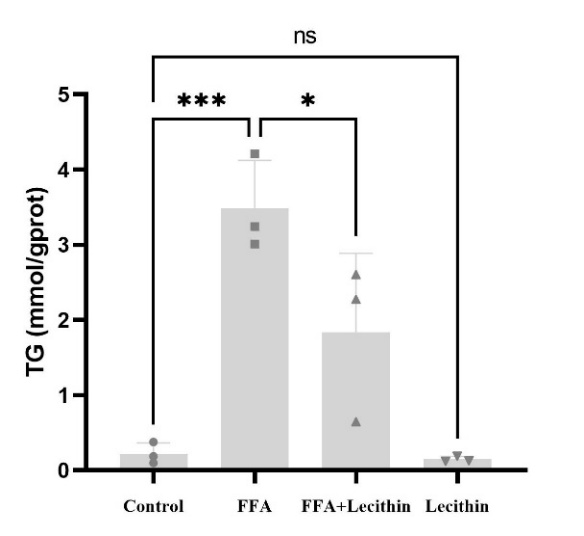


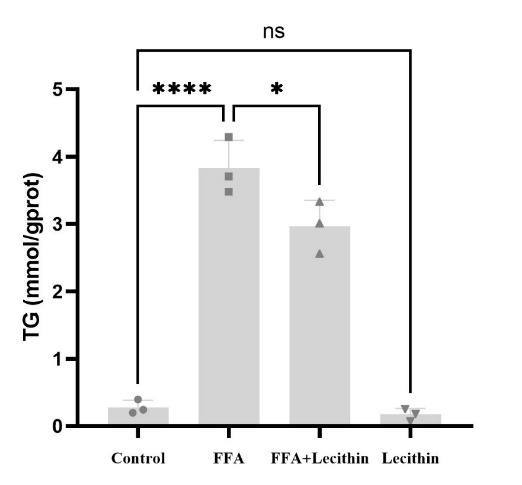


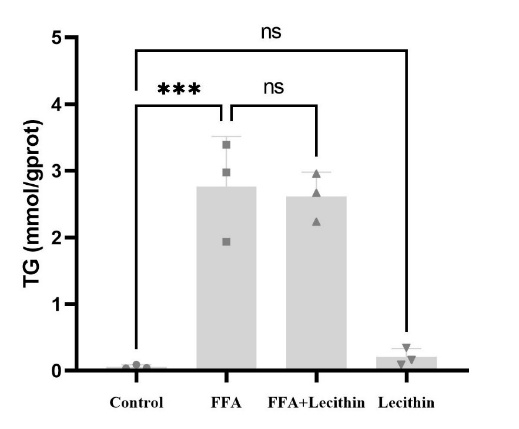


1. stearoyl-CoA desaturase 1 (*SCD1*) expression
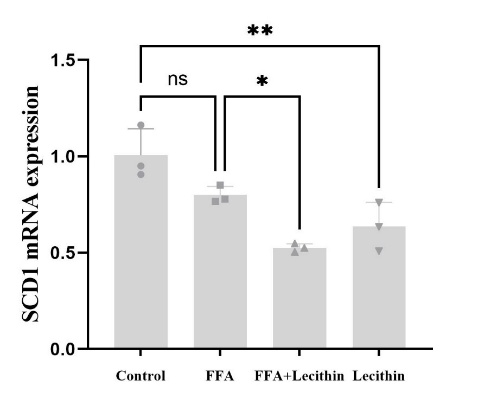


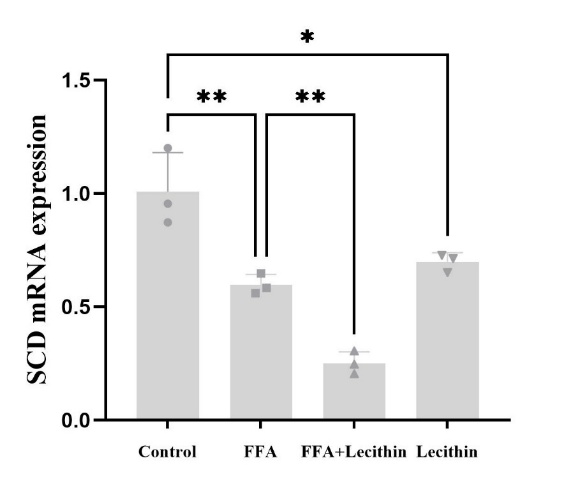


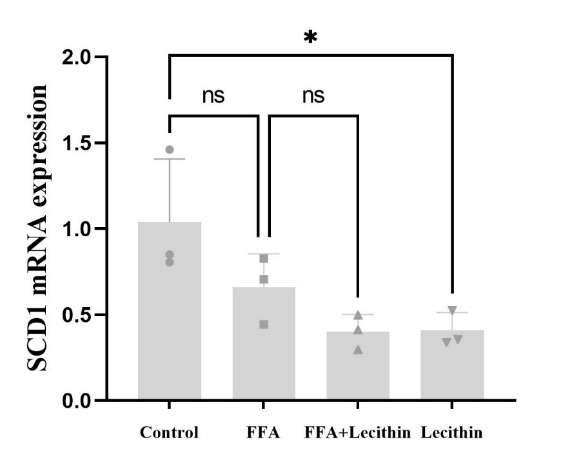


1. acetyl-CoA carboxylase (*ACC*) expression


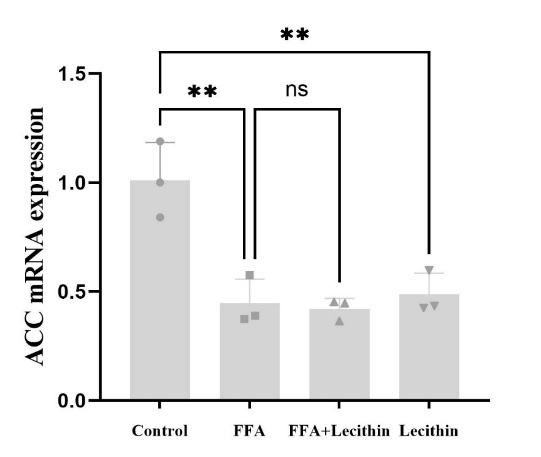


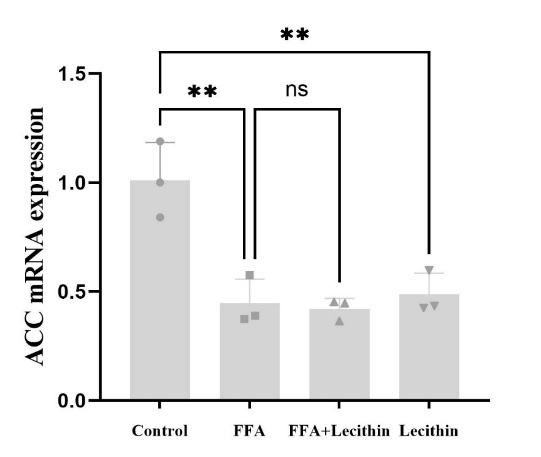


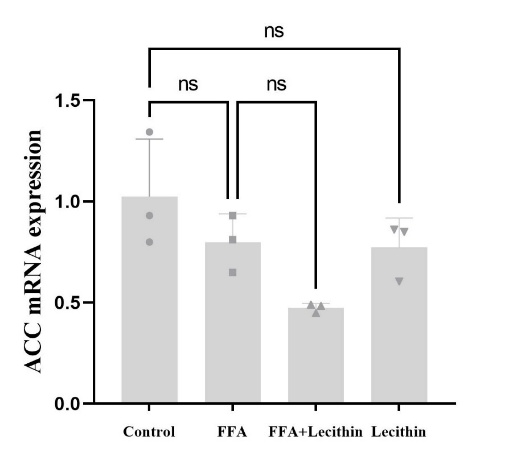

Supplement: Supplementary file 1 [file SupplementaryFile1.docx]
